# Seasonal assessment of drinking water sources in Rwanda using GIS, contamination degree (Cd), and metal index (MI)

**DOI:** 10.1007/s10661-019-7757-9

**Published:** 2019-11-09

**Authors:** Valentine Mukanyandwi, Alishir Kurban, Egide Hakorimana, Lamek Nahayo, Gabriel Habiyaremye, Aboubakar Gasirabo, Theoneste Sindikubwabo

**Affiliations:** 1 Xinjiang Institute of Ecology and Geography, Chinese Academy of Sciences, 818 South Beijing Road, Urumqi, 830011 Xinjiang China; 2 University of Chinese Academy of Sciences, Beijing, 100049 China; 3 University of Lay Adventists of Kigali (UNILAK), P.O. Box 6392, Kigali, Rwanda; 4 Joint Research Center for Natural Resources and Environment in East Africa, P.O. Box 6392, Kigali, Rwanda; 5 Lancaster Environment Centre, Faculty of Science and Technology, Lancaster University, Lancashire, LA1 4YQ UK; 6 Water and Sanitation Corporation, Ltd., P. O. Box 2331, Kigali, Rwanda

**Keywords:** Contamination degree, Metal index, Springs, Water quality, Rwanda

## Abstract

The quality of drinking water source remains as a major concern in areas of developing and underdeveloped countries worldwide. The treatment and supply of drinking water in Rwanda are carried out by Water and Sanitation Corporation, a state-owned public company. However, it is not able to supply water to all households. Consequently, the non-serviced households depend on natural water sources, like springs, to meet their water requirements. Nevertheless, the water quality in these springs is scarcely known. Therefore, this study assessed and compared metal elements in drinking water sources in the dry and rainy seasons in 2017 using the contamination degree, metal index, and geographic information systems to reveal the spatial distribution of water quality within the considered water sources of springs in Rwanda. The samples were collected monthly from nine water sources of springs and the measured elements are aluminium, calcium, copper, iron, manganese, and zinc. The metal index indicated that during the dry season and rainy season, the sites of Kibungo (1.10 and 1.26) and Kinigi (1.01 and 1.54) have assessed a metal index which is higher than 1. Thus, the water quality of those sites was getting the threshold of warning. The analysis indicated that pollutants are easily transported into water bodies during the rainy season in urban and rural areas to a greater extent than during the dry season .

## Introduction

Safe and affordable water is essential for public health. It is used for drinking, food production, domestic use, and recreational purposes. Access to improved water supplies and sanitation, along with better management of water resources, plays a crucial role in developing countries by impacting on communities’ well-being and on national development plans (Onda et al. 2012; Wedgworth and Brown 2013; Nagar et al. 2015). However, access to safe drinking water is limited by global warming, rapid human population growth, inappropriate waste and wastewater management, housing styles, and geographical location. Rural dwellers have much more severe difficulties accessing clean water compared with urban residents (Tiwari et al. 2015; Wedgworth and Brown 2013; Hosseinifard and Aminiyan 2015). The pollution of drinking water sources is gradually increasing, due to limited financial capabilities and poor infrastructure, which force communities to directly consume water from farm wells, springs, and rainwater stores without prior treatment (Bempah and Ewusi 2016; Scott et al. 2017; Sahoo and Patra 2018).

The water quality is compromised by contaminants from anthropogenic sources, including industrial activities and agriculture, and the changing climate creates heavy storms and alters rainfall patterns, which affect the elements found in runoff (Elrashidi et al. 2015; Byer et al. 2011; Neogi et al. 2018). With the growing water demand, efforts have been consolidated to ensure access to safe water, such as creating buffer zone policies controlling the distance for various land uses from water bodies, pollutant reduction, highlighting the role of different stakeholders in water management, water purification, and waste and wastewater treatment (Khan et al. 2015; Juahir et al. 2011).

There has been much attention from local governments, governmental organizations, and NGOs but the burden is still primarily in developing countries (Mdegela et al. 2009). For example, since 1900, 2.6 billion people have gained access to safe water, but inequality still exists. In 2015, 663 million people, representing about 1 in 10 of the world’s population, were drinking unsafe water, and the most affected areas are countries in sub-Saharan Africa (Aboniyo et al. 2017; Allen et al. 2013; Goher et al. 2014; Bidkhori et al. 2018).

In Rwanda, the high population density, expanding industrialization and urbanization, inappropriate waste and wastewater management, high rainfall intensity, and the country’s high elevation are among the key sources of water pollution (Kirby et al. 2016; Nahayo et al. 2016). This pollution is associated with the cost of traveling to and the location of piped water in rural areas, which encourages the residents to use unsafe water. There are also poor sewage systems and common use of public latrines and septic tanks in both rural and urban areas (Aboniyo et al. 2017). Although several scholars (Doyle and Shanahan 2010; Karamage et al. 2016; Nsengimana et al. 2012; Nhapi et al. 2011) have conducted water quality assessments, they lacked the seasonal measurements necessary for understanding temporal changes in water quality, so that appropriate seasonal drinking water source management mechanisms can be applied. Regular water quality assessment would help water resource managers, environmental health officers, and the whole community to better understand and correlate seasonal variability and drinking water quality. Therefore, this study aimed to assess and spatially distribute the concentration of metal elements in drinking water sources between the dry and rainy seasons to determine in which period the pollution of drinking water sources is highest, and suggest appropriate drinking water quality management measures for Rwanda.

## Materials and methods

### Study area

Rwanda is a landlocked country located in Central-East Africa, bordered by Uganda to the north, Burundi to the south, Democratic Republic of Congo to the west and Tanzania to the east (Mukanyandwi et al. 2018). Rwanda has two rainy and two dry seasons, referred to as long and short. The first rainy season runs from March to May and the second spans late September to November, with an average rainfall of 110–200 mm per month. Rwanda also has two dry seasons; the first (short) dry season lasts from December to the end of February, while the longer one begins in June and runs to early September. The average temperature ranges between 19 and 27 °C (Nahayo et al. 2018).

**Fig. 1:**
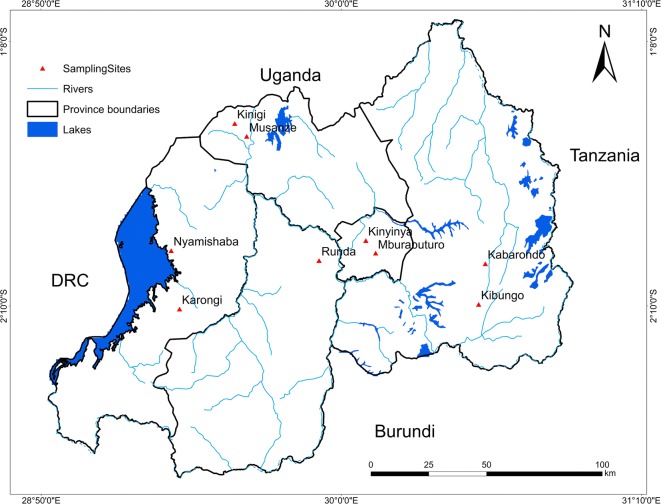
Water sampling locations and the water network in Rwanda

In Rwanda, there are abundant water resources (Fig. 1). The total area of Rwanda is 26,338 square kirometers, including about 128,190 ha of lakes, 7260 ha of rivers, and 77,000 ha are occupied by marshlands. In addition, there are about 22,300 springs (Ali et al. 2014; Nsengimana et al. 2012). To ensure that water undergoes prior treatment and to increase access to safe drinking water, about sixteen water treatment plants are operating countrywide. These initiatives have increased the percentage of people accessing safe drinking water from 23% in 1990 to 82% in 2016 (Rutanga and Niyigena 2016; Karamage et al. 2016) . Therefore, The water samples were collected from springs in different areas in country to ensure that the water quality of selected springs is  healthy according to the World Health Organization for metals in water(Table 1).

**Table 1:** Names and location of the sampling sites

No	Sampling sites	Location (province)
1	Kinyinya	Urban, Kigali city
2	Mburabuturo	Urban, Kigali city
3	Runda	Semi-Urban, Southern Province
4	Kabarondo	Semi-Urban, Eastern Province
5	Kibungo	Rural Eastern Province
6	Musanze	Semi-urban, Northern Province
7	Kinigi	Rural, Northern Province
8	Karongi	Semi-urban, Western Province
9	Nyamishaba	Rural Western Province

### Field sample collection and experimental approaches

The water samples were collected from nine drinking water sources located in urban, semi-urban, and rural areas of Rwanda, during the dry season (July–September) and rainy season (October–December), respectively.

The authors collected three water samples monthly from each sampling site from a foot below the water surface. The samples were preserved in acid-washed 100-mL polypropylene bottles to avoid changes in characteristics, and were digested, concentrated, and prepared for analysis by atomic absorption spectrophotometry (AAS) using an AA Spectrometer M Series.

**Fig. 2:**
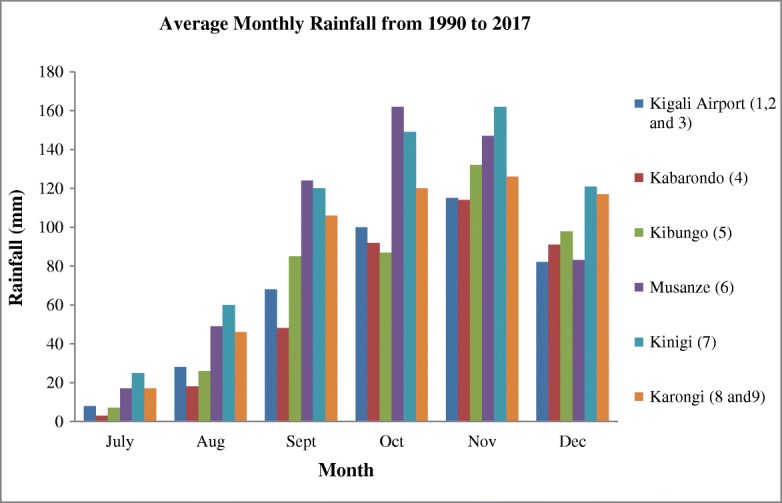
Average monthly rainfall recorded at meteorological stations neighboring the water sampling sites

We conducted extensive monthly water quality monitoring and the measured elements were calcium (Ca), iron (Fe), manganese (Mn), copper (Cu), aluminium (Al), and zinc (Zn). The drinking water samples of dry and rainy seasons were compared, the monthly maximum rainfall (mm) records at each meteorological station neighboring the water sampling site were obtained (Fig. 2) for July, August, and September for the dry season, and October, November, and December for the rainy season of 2017. During the analysis, the rainfall variability was referred to in order to better understand how changes in rainfall might influence the drinking water quality.

### Indexing approach

#### Metal index

This study used the metal index (MI); it is generally used for metals in drinking water quality (Goher et al. 2014). The metal index takes into account possible additive effect of metal elements on the human health that help to quickly evaluate the overall quality of drinking waters. Metal index is given by the expression proposed by Caeiro et al. (2005) and is calculated as indicated below:

1 $$ MI=\sum \limits_{i=1}^n\frac{CI}{\left(\mathrm{MAC}\right)i} $$

where MAC is the maximum allowable concentration and Ci is the mean concentration of each element. The higher concentration of metal was compared with its respective MAC value that shows the worse quality of water. MI value > 1 is a threshold of warning (Bakan et al. 2010). Water quality and its suitability for drinking purpose can be examined by determining its metal pollution index (Mohan et al. 1996; Reza and Singh 2010). This study applied the above metal index for the estimate value of six metal elements, namely, aluminium, calcium, manganese, copper, iron, and zinc.

#### Contamination degree (Cd)

The contamination degree is defined as the sum of all concentration factors (Rahman et al. 2014). The water samples are classified by calculating the degree of contamination in water samples. Contamination degree, by added various parameters assuming water quality, investigates the convenience of drinking water samples for municipal consuming (Backman et al. 1998). The contamination degree has to be calculated split up for all samples, based on the surpassed parameters from standard values. This index is calculated as follows:

2 $$ \mathrm{Cd}=\sum \limits_{i=1}^n{\mathrm{Cf}}_{\mathrm{i}} $$

Cf_i_ can be obtained from Eq. (3)

3 $$ {\mathrm{Cf}}_{\mathrm{i}}=\frac{{\mathrm{CA}}_{\mathrm{i}}}{{\mathrm{CN}}_{\mathrm{i}}}-1 $$

Where:

Cf_i_: is the contamination factor for the *i*th parameter
CA_i_: is the measured value for the *i*th parameter
CN_i_: is the standard allowed value for the *i*th parameter

## Result

The results illustrated in Tables 2 and 3 indicated gradual increase of the values of metal elements, particularly during the dry season (July–September 2017) compared with the values noted during the rainy season (October–December 2017). The mean concentrations of the analyzed metals were used to calculate the metal index (MI) and contamination degree (Cd) during both seasons and the mean concentration of elements on sites were used to calculate the metal index and contamination degree of each sampling site.

**Table 2:** Concentration and mean value of metal elements during the dry season

Sites	1	2	3	4	5	6	7	8	9	Mean/element
Ca	43.6	54	61.4	31	91.8	63.2	84	21	26	52.88
Fe	0.38	1.14	0.2	0.39	0.33	0.09	0.2	0.2	0.09	0.33
Mn	0.4	0.21	0.02	0.17	0.32	0.01	0.4	0.003	0.11	0.18
Cu	0.51	0.28	0.13	1.31	1.1	0.18	0.03	0.41	0.12	0.45
Al	0.03	0.11	0.03	0.16	0.03	0.12	0.11	0.08	0.09	0.08
Zn	0.38	0.38	0.4	0.54	0.37	0.10	1.01	0.11	1	0.47
Mean/site	7.55	9.35	10.36	5.59	15.65	10.61	14.29	3.63	4.56

**Table 3:** Concentration and mean value of metal elements during rainy season

Sites	1	2	3	4	5	6	7	8	9	Mean/element
Ca	75.2	92	82	72.8	102.7	77.1	127.1	37.8	32.1	77.46
Fe	0.71	1.24	0.61	0.43	0.65	0.07	0.08	0.18	0.13	0.45
Mn	0.16	0.26	0.14	0.32	0.44	0.2	0.15	0.04	0.14	0.2
Cu	0.65	1.46	0.8	1.78	1.9	0.2	1.06	0.84	1.17	1.09
Al	0.09	0.32	0.06	0.28	1.01	0.15	0.86	0.14	0.19	0.34
Zn	0.8	0.83	0.9	0.76	0.58	1.13	1.08	0.14	1.04	0.79
Mean/site	12.93	16.01	14.08	12.72	17.88	13.14	21.72	6.52	5.79	

Table 4 illustrates the metal index and the contamination degree of measured metal elements in drinking water sources.

**Table 4:** The metal index and contamination degree of measured elements during the dry season (ds) and rainy season (rs)

Metal elements	Ci or CAi in ds (mg/L)	(MAC)i or (CNi)/mg/L	Ci or CAi in rs	MI (ds)	MI (rs)	Cd (ds)	Cd (rs)
Ca	52.88	80.0	77.46	0.66	0.92	− 0.34	− 0.08
Fe	0.33	0.3	0.45	1.1 (threshold of warning)	1.49 (threshold of warning)	0.1	0.49
Mn	0.18	0.1	0.2	1.8 (threshold of warning)	2.00 (threshold of warning)	0.8	1.00
Cu	0.45	1.0	1.09	0.45	1.09 (threshold of warning)	− 0.55	0.09
Al	0.08	0.2	0.34	0.40	1.70 (threshold of warning)	− 0.60	0.70
Zn	0.47	3.0	0.79	0.15	0.26	− 0.85	− 0.74

The table below represents the metal index for each drinking water sampled during both seasons and it also indicates the sites which are on a threshold level of warning according to their recorded metal index value (Table 5).

**Table 5:** Metal index and contamination degree of each sampling site

Sampling sites	Cd	Cd	MI	MI
	Dry season	Rainy season	Dry season	Rainy season
1	− 0.46	− 0.08	0.53	0.91
2	− 0.33	0.13	0.66	1.13 (threshold of warning)
3	− 0.26	0.001	0.73	0.99
4	− 0.6	− 0.09	0.39	0.90
5	0.1	0.27	1.10 (threshold of warning)	1.26 (threshold of warning)
6	− 0.24	− 0.06	0.75	0.93
7	0.01	0.54	1.01 (threshold of warning)	1.54 (threshold of warning)
8	− 0.74	− 0.53	0.25	0.46
9	− 0.67	− 0.58	0.32	0.41

According to Caeiro et al. (2005) and Lyulko et al. (2001), the classification of metal index to the drinking water quality classifies into six different classes and their characteristics as illustrated in Tables 6 and 7.

**Table 6:** Classification of metal index

MI	Characteristics	Class	Site no. in dry season	Site no. in rainy season
< 0.3	Very pure	1	8	–
0.3–1.0	Pure	2	1, 2, 3, 4, 6, 7, and 9	1, 3, 4, 6, 8, and 9
1.0–2.0	Slightly affected	3	5	2, 5, and 7
2.0–4.0	Moderately affected	4	–	–
4.0–6.0	Strongly affected	5	–	–
> 6.0	Seriously affected	6	–	–

**Table 7:** Water quality classification using contamination degree (Cd)

Cd	Characteristics	Class	Sampling site
Cd < 1	Low contamination	1	All sampling site
1 < Cd	Moderate contamination	2	
Cd > 3	High contamination	3	

The contamination degree classifies drinking water quality into three different classes as illustrated in the Table 7; it revealed that all sampled sites were classified in class 1 which is characterized by low contamination where the Cd < 1 (Table 6).

Figures 3, 4, 5, 6, 7, 8, 9, and 10 represent the spatial distribution of each metal element during the dry and rainy seasons.

**Fig. 3:**
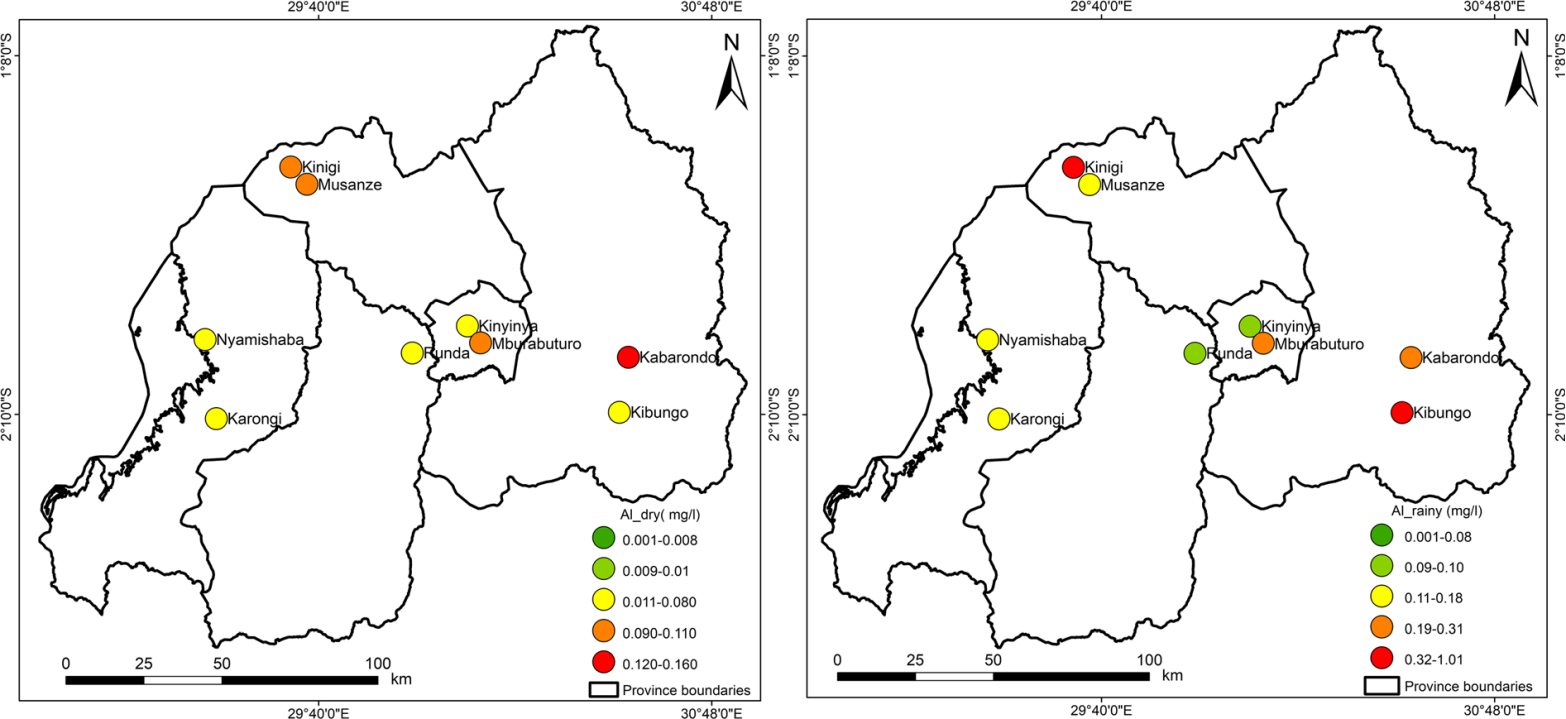
Spatial distribution of aluminium during the dry and rainy seasons

**Fig. 4:**
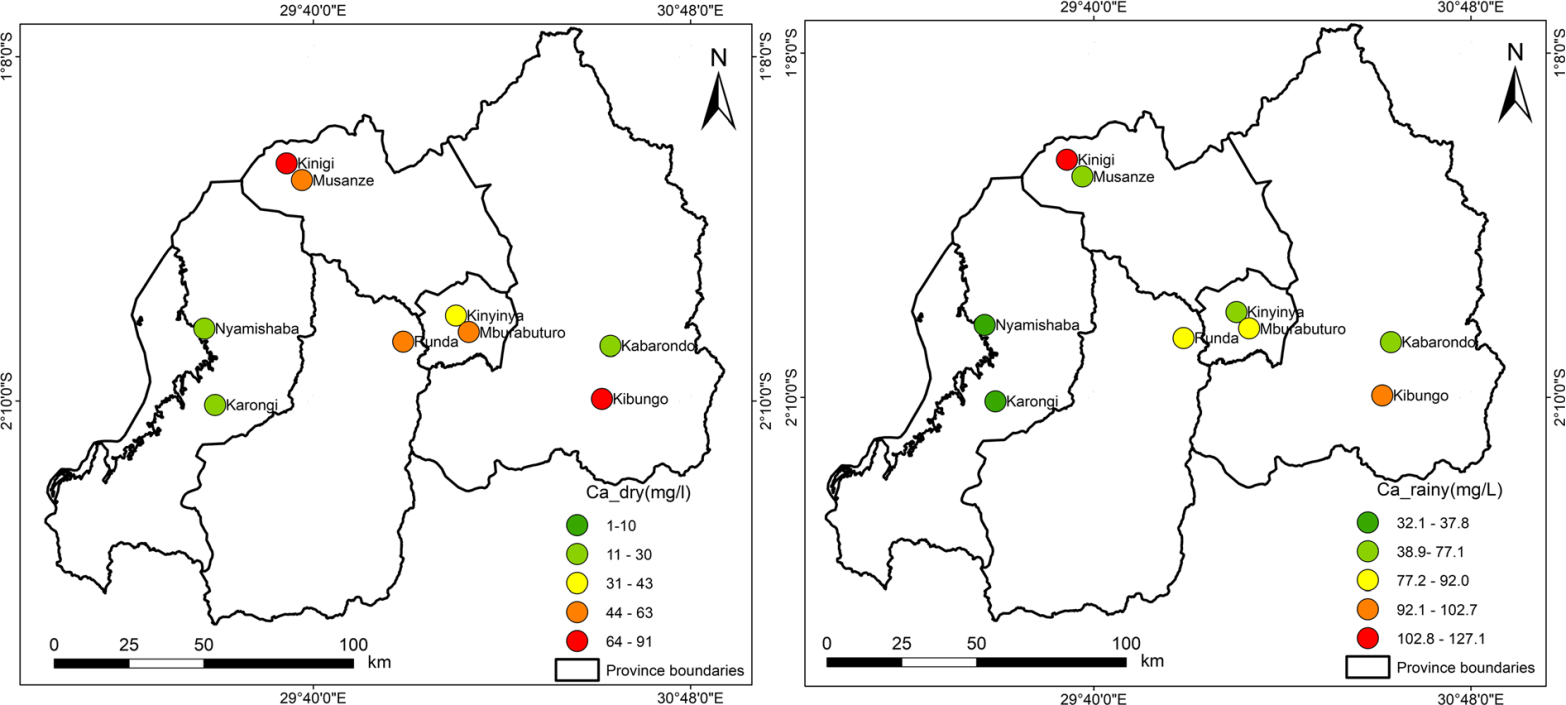
Spatial distribution of calcium during the dry and rainy seasons

**Fig. 5:**
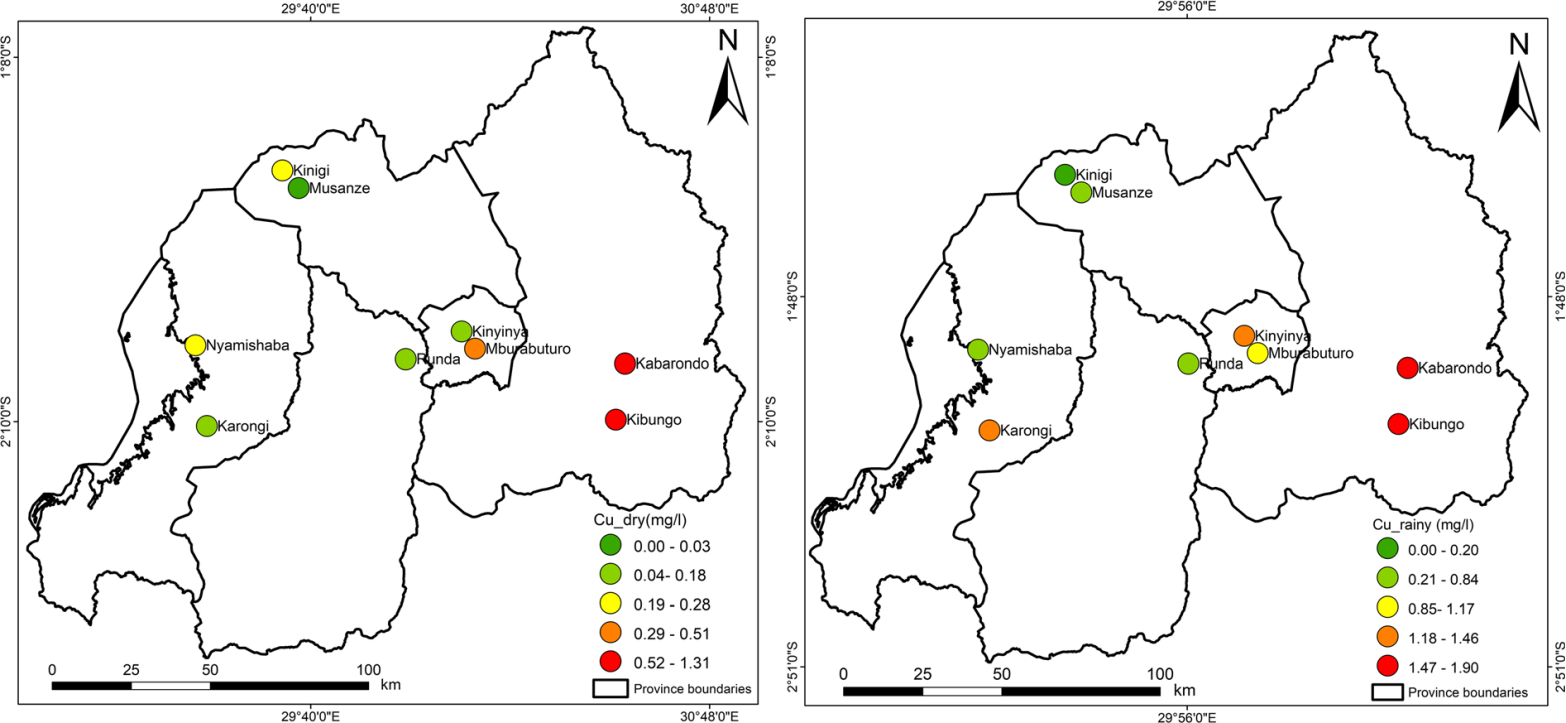
Spatial distribution of copper during the dry and rainy seasons

**Fig. 6:**
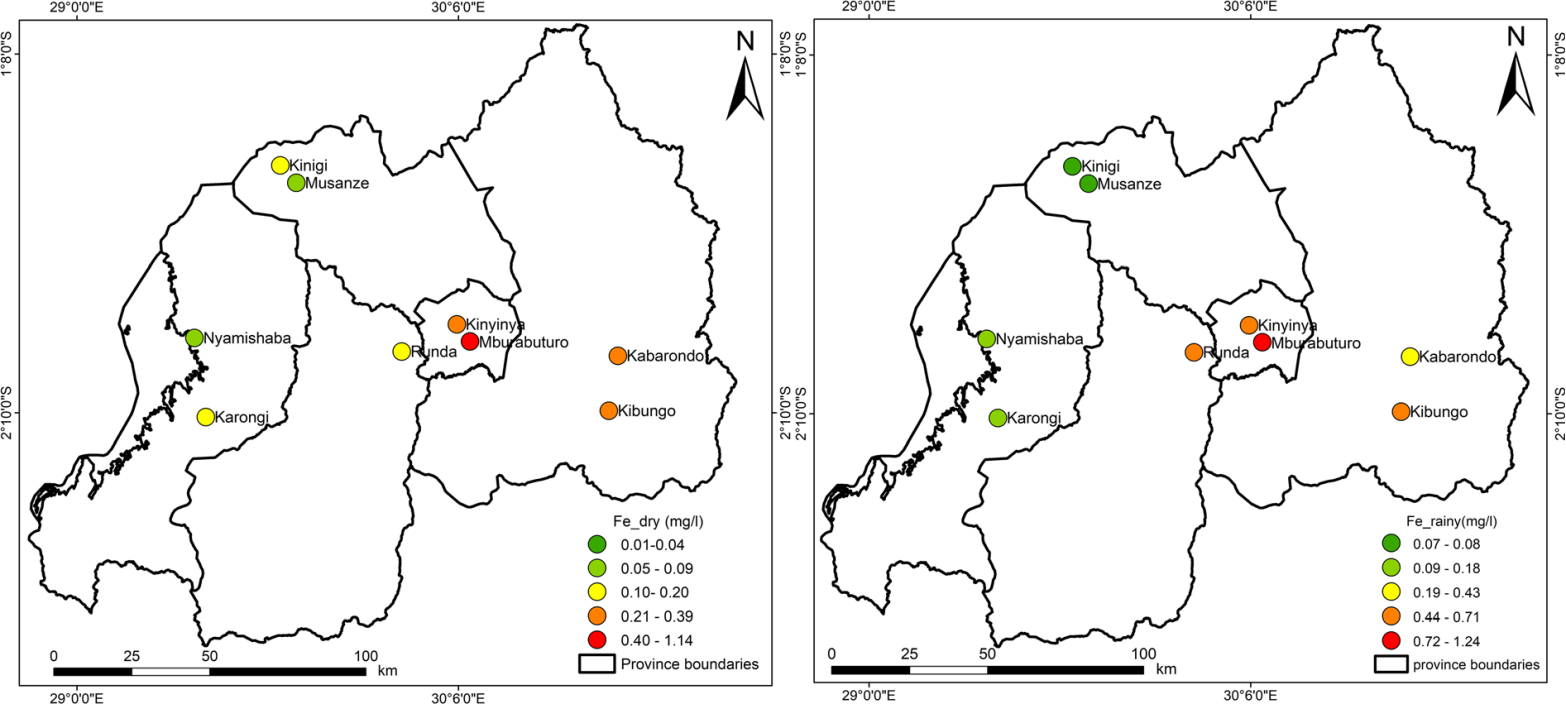
Spatial distribution of iron in the dry and rainy seasons

**Fig. 7:**
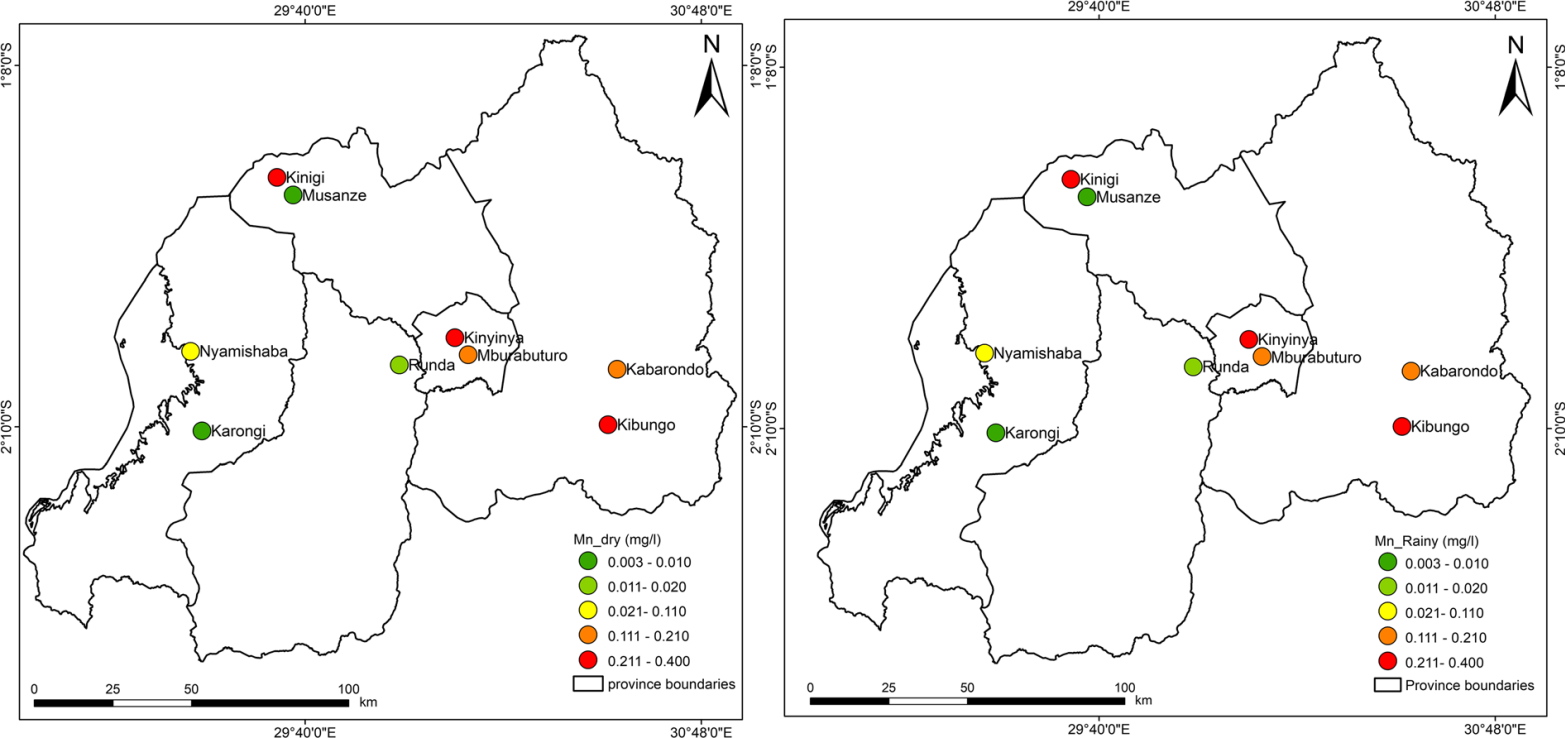
Spatial distribution of manganese during the dry and rainy seasons

**Fig. 8:**
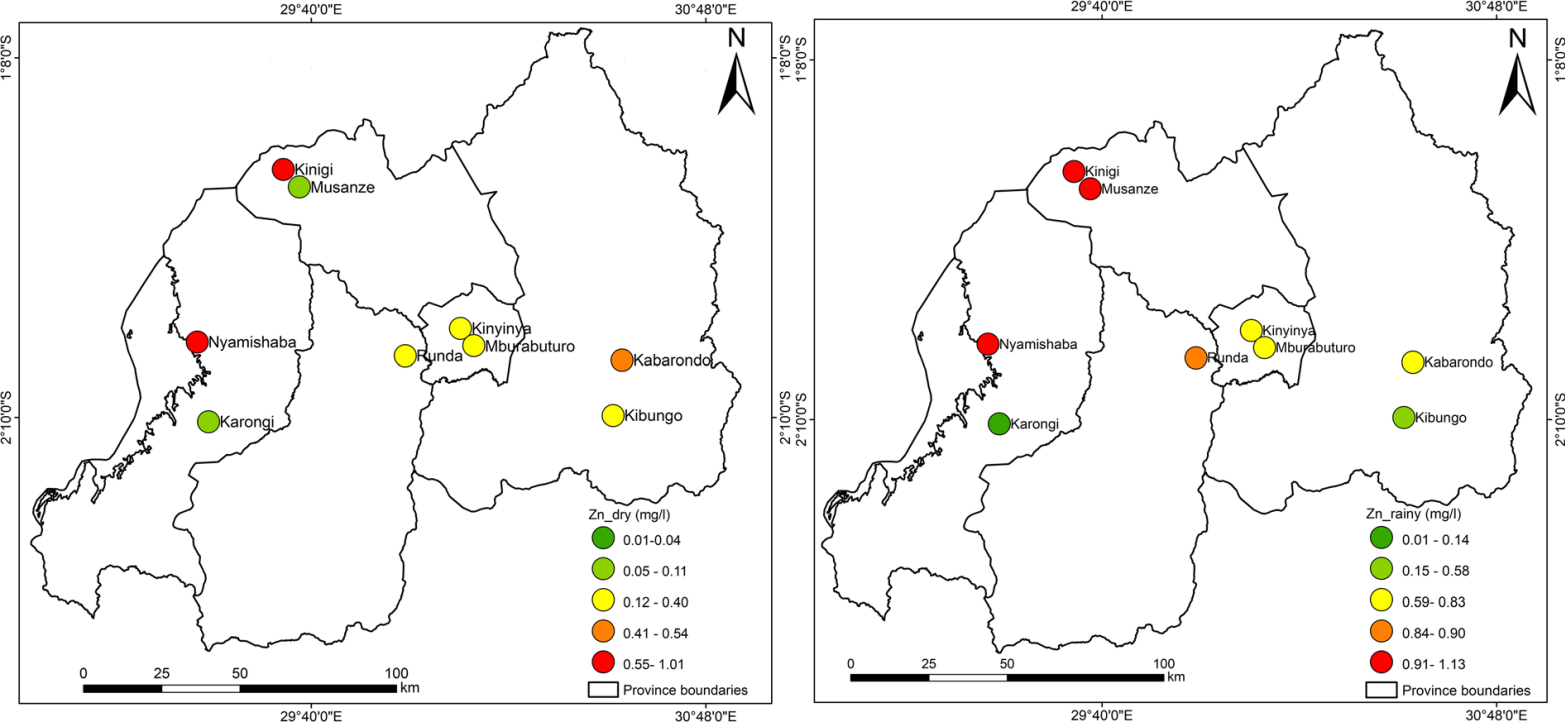
Spatial distribution of zinc in the dry and rainy seasons

**Fig. 9:**
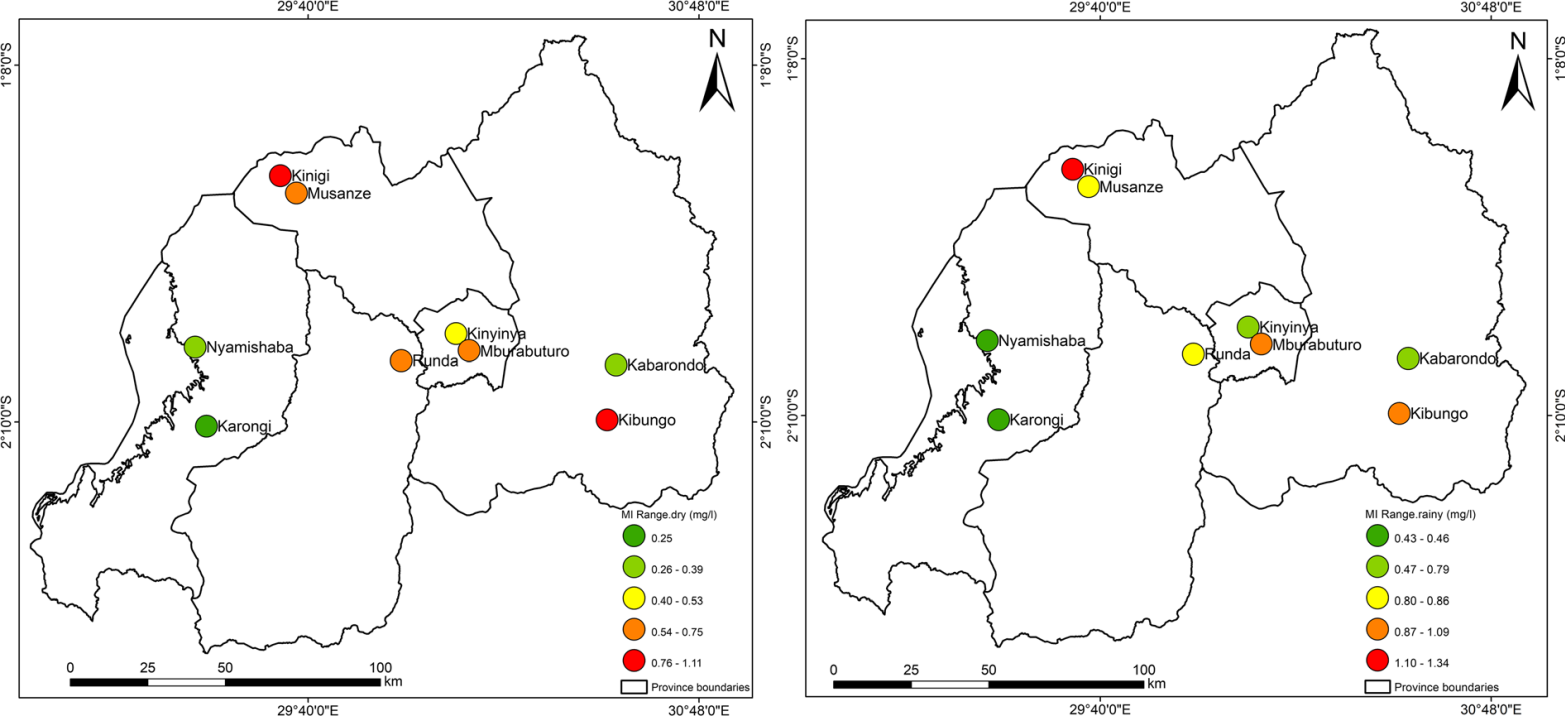
Spatial distribution range of metal index (MI) for each sampling site

**Fig. 10:**
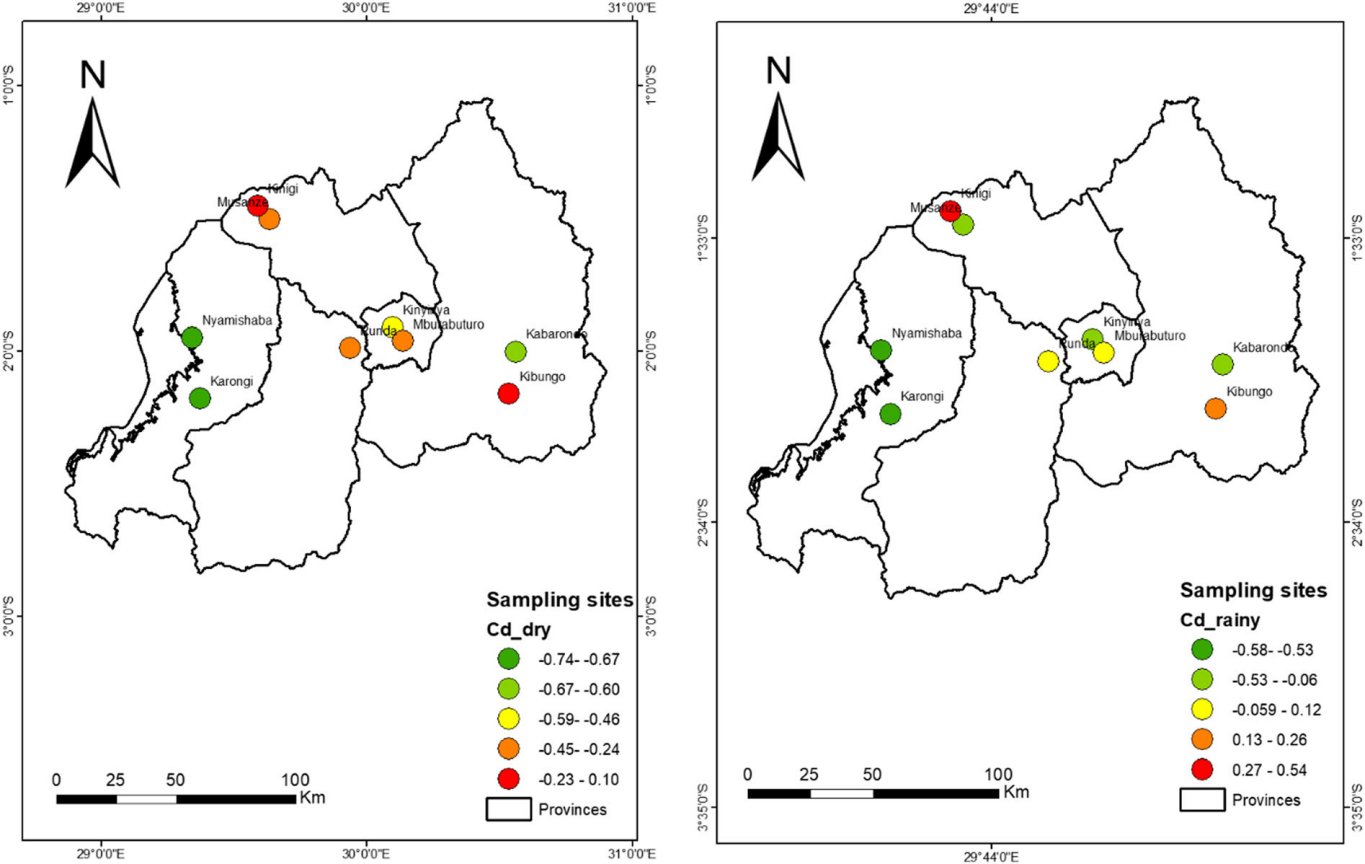
Spatial distribution range of contamination degree (Cd) for each sampling site

## Discussions

As shown in Table 5 and Fig. 2, the sampling sites located in areas with high rainfall similarly recorded higher metal pollution index and higher contamination degree. This impact of rainfall patterns on water quality is marked by the results of this study; the rainfall acted effortlessly as a driver to water pollution, where high MI was recorded during the rainy season than MI during the dry season. The metal index and contamination degree per each sampling site indicated the high value of index during the rainy season at the sampling sites of Mburabuturo, Kibungo, and Kinigi compared with the metal index and contamination degree during the dry season, but also higher at the sampling site of Kibungo and Kinigi at large extent than other sampling sites. The maximum metal index was assessed at Kinigi site (1.34) during the rainy season and at Kibungo site (1.1) during the dry season (Table 5). Contingent on classification of metal index for drinking water quality, the average index for samples of dry and rainy seasons was 0.63 and 0.94.

According to metal index’s classification on water quality, during the dry season, most of the sites are classified in class 2 which is characterized as pure, while site 8 is classified in class 1 (very pure). Besides, site 5 is classified as slightly affected (class 3). The sites of 1, 3, 4, 6, 8, and 9 are classified in class 2 (pure), whereas the sites 2, 5, and 7 are classified as slightly affected in class 3 (Table 6).

The maximum degree of contamination was recorded for the site of Kinigi (0.54) during the rainy season, while during the dry season, the maximum contamination degree was 0.1 for Musanze site; the minimum degree of contamination has been fixed for the Karongi site (− 0.74) in the dry season and (− 0.58) in the rainy season at Nyamishaba site (see Table 5). The contamination degree average for all the sites during the dry and rainy seasons is − 0.35 and − 0.04, which are classified in class 1 as low degree of contamination.

In this study, drinking water sources located in urban areas, such as site of Kinyinya and Mburabuturo are polluted compare with the sampling sites located in rural areas. This agrees with previous reports (Srivastava et al. 2011; Lumb et al. 2011; Nyangababo et al. 2005) on water quality monitoring, which highlighted how urban waters are predominantly becoming polluted at high extent compared with that located in rural areas due to wastes generated by households, industries, slaughtering houses, directly thrown into waters, and other urban activities located nearby water bodies. Although the proportion of people accessing on safe drinking water increased in the last years in Rwanda, the results of this study (Fig. 8) showed that some people still consume polluted water, mostly during the rainy season than in the dry season. The iron and manganese are the key pollutants in the drinking water sources considered in this study (Table 5). Accordingly, it was observed that the consumers of polluted water sources might be subject to toxicity of the nervous system and cancer, liver, heart, and pancreatic damage as a result of excess manganese and iron, the highly noticed pollutants among other measured elements. Therefore, this expresses how rainfall undermines water quality as it facilitates easy pollutants runoff downwards water bodies. But also, on the other side, it calls for urgent rainfall management, such as rainfall harvest and/or use of bench terraces and agroforestry on hills surrounding the water bodies to minimize the runoff. This to be successful, it requires appropriate seasonal water quality management measures, specifically during the rainy season in urban areas than in rural areas to minimize the pollution likelihood of the available water sources (Fig. 1), which, in turn, affects water consumers’ health. Furthermore, this will reduce the cost of water treatment and enhance community’s wellness and natural resources management as well. Many researchers focused on seasonal evaluation of the assessment of metals in lakes and rivers (Low et al. 2016; Mukanyandwi et al. 2018; Rajeshkumar et al. 2018) but they did not work on the springs which are used with numerous people especially in developing countries. The accessibility of water supply is still inadequate in Rwanda; more than 38% of Rwandan people use natural sources including springs and the information about water quality of those springs was unknown. However, this study was contributed to assess and spatially distribute the metal concentration in drinking water by measuring in which season the drinking water is more polluted and to which extent the drinking water sources are polluted based on indices according to the drinking water quality standard.

## Conclusion

Water quality and scarcity are widespread problems and its sustainable management is becoming a quite challenge. Even though a range of resolving suggestions have been provided such as provision of investment in water infrastructure maintenance, water reuse, recycle, flotation, chemical precipitation, ion exchange and membrane filtration and coagulation-flocculation, the rapid human population growth, and increase on-point and non-point drinking water pollution sources are threats to water quality. The government of Rwanda has launched the Integrated Water Resources Management (IWRM), an approach of developing, monitoring, and managing water resources. Nonetheless, for the policy to be fruitful and sustainable, there is a great need of managing the wastewater, the rapid expanding urbanization and informal settlements, and industrial and mining activities.

Therefore, this study employed metal index and contamination degree to compare the drinking water quality sources during the rainy and dry seasons by measuring for which extent the drinking water sources used are polluted. GIS has been used to spatially distribute the seasonal concentration of metal elements of drinking water sources in Rwanda. The results indicated that the mean value of iron and manganese exceeded the drinking water guidelines of the World Health Organization than other elements measured; also these elements were highly indexed than other monitored elements.

Therefore, the following are suggested for the water quality management in Rwanda under the above-mentioned threats of water quality in Rwanda:

1. Since Rwanda is rich in precipitation throughout the year, it is good to consider maximum rain harvest; this will increase the underground storage and enables local communities to supply drinking water to their infrastructure and reduces sediments carried into source water which are the sources of metals in water.
2. The bench terraces and agroforestry techniques must be applied to minimize the sediments runoff.
3. It is suggested to promote environmental research and education and training on drinking water sources management from basic schools, hydrological data sharing and free access for water quality management enhancement, and to supervise the implementation of the buffer zones policy under execution in Rwanda.
4. Rapid population growth is increasingly leading to natural resource degradation; it is advised to set a fixed number of children per family with penalties or tax incentives to those disregarding the policy.
5. Population growth requires sufficient food, to do so, irrigation is proposed to boost the agricultural production; however, it is good to first check on environmental pros and cons of every irrigation technique (sprinkler and flood irrigation, drip irrigation) before use.
6. Rwanda as a developing country with high water demand, water re-use, the desalination would help much, where industrial, saline water, and household wastewater can be turned into usable water for other uses such as garden watering, carwash, and toilet uses. This will be a good option and reduce the wastewater-associated consequences to water quality.
7. Although the government prioritized Crop Intensification Program (CIP), with one crop at appropriate location, it would be good to initiate and promote break crop system, different crops at once; this will enhance soil fertility and maintain soil at a level of not demanding high chemical fertilizers, and reduces water pollution.
8. Even though, environmental management is a cross cutting issue at every decision-making level, monitoring and evaluation of its execution and success basing on community’s reality and national development plans are highly suggested.
